# Transcriptomic Insight into Terpenoid Biosynthesis and Functional Characterization of Three Diterpene Synthases in *Scutellaria barbata*

**DOI:** 10.3390/molecules23112952

**Published:** 2018-11-12

**Authors:** Huabei Zhang, Baolong Jin, Junling Bu, Juan Guo, Tong Chen, Ying Ma, Jinfu Tang, Guanghong Cui, Luqi Huang

**Affiliations:** 1 State Key Laboratory Breeding Base of Dao-di Herbs, National Resource Center for Chinese Materia Medica, China Academy of Chinese Medical Sciences, Beijing 100700, China; zhb1050217242@163.com (H.Z.); jblhandan@163.com (B.J.); bujl003@163.com (J.B.); guojuanzy@163.com (J.G.); chentong_biology@163.com (T.C.); xiaoma1110@126.com (Y.M.); jinfutang@126.com (J.T.); 2 College of Pharmacy, Hubei University of Chinese Medicine, Wuhan 430065, China

**Keywords:** *Scutellaria barbata*, transcriptomics analysis, terpenoid backbone biosynthesis, terpene synthase, diterpene synthase

## Abstract

*Scutellaria barbata* (Lamiaceae) is an important medicinal herb widely used in China, Korea, India, and other Asian countries. *Neo*-clerodane diterpenoids are the largest known group of *Scutellaria* diterpenoids and show promising cytotoxic activity against several cancer cell lines. Here, Illumina-based deep transcriptome analysis of flowers, the aerial parts (leaf and stem), and roots of *S. barbata* was used to explore terpenoid-related genes. In total, 121,958,564 clean RNA-sequence reads were assembled into 88,980 transcripts, with an average length of 1370 nt and N50 length of 2144 nt, indicating high assembly quality. We identified nearly all known terpenoid-related genes (33 genes) involved in biosynthesis of the terpenoid backbone and 14 terpene synthase genes which generate skeletons for different terpenoids. Three full length diterpene synthase genes were functionally identified using an in vitro assay. *Sb*TPS8 and *Sb*TPS9 were identified as normal-CPP and *ent*-CPP synthase, respectively. *Sb*TPS12 reacts with *Sb*TPS8 to produce miltiradiene. Furthermore, *Sb*TPS12 was proven to be a less promiscuous class I diterpene synthase. These results give a comprehensive understanding of the terpenoid biosynthesis in *S. barbata* and provide useful information for enhancing the production of bioactive *neo*-clerodane diterpenoids through genetic engineering.

## 1. Introduction

**Figure 1:**
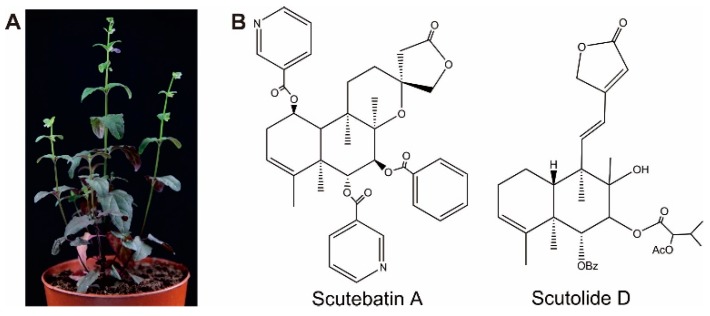
The major medicinally active diterpenoids in *S. barbata*. (**A**) *S. barbata* plant. (**B**) The typical *neo*-clerodane diterpenoids scutebatin A and scutolide D.

The Lamiaceae family produces abundant diterpenoids. Abietanes, labdanes, *neo*-clerodanes, primaranes, and *ent*-kaurenes are the most common diterpenoids in this family [1]. Diterpenoids usually are taxa-specific, for example, the majority of *neo*-clerodanes have been found in the subfamilies Scutellarioideae and Ajugoideae, especially in the genus *Scutellaria*, which has 98 different types [2]. *Scutellaria* has around 360 species, is cosmopolitan in distribution, occurs in a wide range of habitats, and includes annual and perennial herbs and shrubs [3]. In China, two species, *S. barbata* (Figure 1A) and *S. baicalensis* are recorded in the Chinese Pharmacopoeia [4], and more than 150 *neo*-clerodane diterpenoids have been isolated from species in this genus. These *neo*-clerodane diterpenoids have promising cytotoxic activity against several cancer cell lines [5,6,7,8,9]. Scutebatin A and Scutolide D (Figure 1B) are two typical metabolites isolated from *S. barbata* that have significant inhibitory effects on lipopolysaccharide-induced nitric oxide production [10] and inhibition against Epstein–Barr virus lytic replication [11].

In addition to the general characteristics of diterpenoids in Lamiaceae as a whole, they are also especially renowned for essential oils only common in the subfamily Nepetoideae [1]. The volatile oils in this subfamily mainly consist of monoterpenoids and sesquiterpenoids. Iridoids, which are monoterpenoid lactones, are also produced in most species of the Lamioideae, Viticoideae, Ajugoideae, and Scutellarioideae. A recent study investigated the diversity mechanism of the three terpenoid classes (monoterpenes, sesquiterpenes, and iridoids) in Lamiaceae [12]. It showed that multiple mechanisms contributed to the evolution of chemodiversity. Gene family expansion rather than increased enzyme promiscuity of terpene synthases is correlated with mono- and sesquiterpene diversity and differential expression of core genes within the iridoid biosynthetic pathway is associated with iridoid presence/absence. However, besides the diterpenoids reported from *S. barbata,* little is known about the monoterpenes and sesquiterpenes in *S. barbata*.

Despite their structural diversity, all terpenoids begin with the five-carbon building blocks isopentenyl pyrophosphate (IPP) and its isomer dimethylallyl pyrophosphate (DMAPP). In plants, both IPP and DMAPP are synthesized by two independent pathways, the mevalonic acid (MVA) and methylerythritol phosphate (MEP) pathways. However, metabolic cross-talk via the exchange of IPP and to a lesser extent DMAPP is observed between these two pathways in both directions [13,14,15]. Recently, an alternative MVA pathway was found. These studies showed that isopentenyl phosphate kinase (IPK) can reactivate isopentenyl phosphate (IP) via ATP-dependent phosphorylation, thus forming the primary metabolite IPP and altering conventional views of metabolic regulation of terpenoid metabolism in plants [16,17,18]. IPP and DMAPP are subsequently used in multiple compartments by short-chain prenyltransferases (GPP, FPP, and GGPP synthases) to produce prenyl diphosphate intermediates, including geranyl diphosphate (GPP, C10), farnesyl diphosphate (FPP, C15), and geranylgeranyl diphosphate (GGPP, C20). These precursors are further synthesized by a family of terpene synthases (TPSs) to form 10-carbon monoterpenes, 15-carbon sesquiterpenes, and 20-carbon diterpenes.

Labdane-related diterpenoids are typically formed from GGPP via the sequential action of two diterpene synthases [19]. The first enzyme, a class II diTPS (TPS-c subfamily) carries out a protonation-initiated cyclization of GGPP to generate the characteristic decalin core; it also initially establishes the stereochemistry of the decalin core (e.g., *ent*, *syn*, normal/(+)). The second enzyme, a class I diTPS (usually from the TPS-e/f subfamily), then ionizes the cyclic intermediate by removal of the diphosphate group and often guides further carbocation-driven rearrangements. Diterpenoid biosynthesis has been thoroughly investigated in 10 species from subfamilies Nepetoideae [20,21,22,23,24,25,26,27,28,29,30,31,32], Lamioideae [33], and Viticoideae [34]. However, no diTPS synthases have been reported from Scutellarioideae.

Here we report nearly all known genes involved in terpenoid biosynthesis from *S. barbata*. We established a de novo transcriptome assembly of *S. barbata* using flower, aerial parts (stem and leaves), and root. Through sequence homology-based transcriptome annotation, we identified 33 genes involved in terpenoid backbone biosynthesis, and 14 terpene synthase genes. Furthermore, three diTPS genes were functionally identified. This study, representing the first transcriptomic resource for *S. barbata*, gives an overview of terpenoid biosynthesis in *S. barbata*, and lays a foundation for future studies on the functional characterization of candidate genes involved in the biosynthesis of pharmacologically active *neo*-clerodane diterpenoids in *S. barbata*.

## 2. Results

### 2.1. Transcriptome Sequencing, Assembly, and Annotation

**Figure 2:**
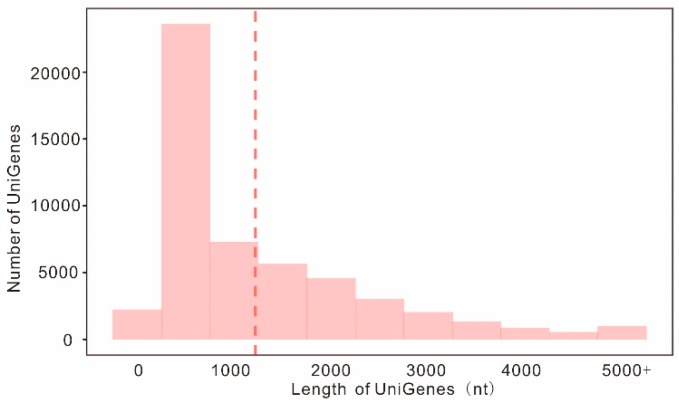
Length distribution of assembled genes. Genes with lengths larger than 5 kb are counted together. The dashed line represents average length of all unigenes.

Since no genomic or transcriptomic resources for *S. barbata* were available, we performed RNA-seq based deep transcriptome profiling to derive a de novo transcriptome assembly. In order to achieve complete representation of the *S. barbata* transcriptome, total RNA was extracted from flowers, aerial parts, and root (Figure S1; Table S1). Individual libraries for each sample were sequenced using the Illumina HiSeq™2000 platform in paired-end mode, resulting in a total of 237,517,352 strand-specific paired-end reads with lengths around 150 nt (35,627,602,800 bases) (Figure S2; Table S2). In total, 88,980 transcripts were obtained with an average length of 1370 nt and an N50 length of 2144 nt, indicating high assembly quality; these isotigs were further grouped into 52,211 unigenes, and there were 22,421 (42.94%) unigenes longer than 1000 bp (Figure 2; Table S3). Those assemblies were routinely annotated based on protein similarity using BLAST searches against several public databases, such as Swiss-Prot, TrEMBL, Pfam (HMM), Gene Ontology, and the Kyoto Encyclopedia of Genes and Genomes (Figures S3 and S4). Taken together, 36,659 unigenes (70.23%) were assigned at least one putative function from one of these databases.

### 2.2. Identification of 33 Unigenes Involved in Terpenoid Backbone Biosynthesis

Based on the integrated annotation process, 71 unigenes were annotated as genes involved in terpenoid backbone biosynthesis (Table S4), which showed that similarity-based BLASTX searching using Swiss-Prot database was a more powerful approach than other methods. After further manual annotation by searching the NCBI non-redundant protein sequence database using BLASTX method, we found 27 unigenes with high similarity to genes in bacteria or animals, and those genes were all expressed in root or flower with very low expression levels (Table S4), which may be caused by contamination of microorganisms in those organs. Two unigenes (TRINITY_DN82648_c0_g1 and TRINITY_DN82648_c0_g3) were annotated as different parts of one gene. Six unigenes shorter than 500 bp, together with four unigenes did not match the criteria for functional annotation. Thus, we found 33 unigenes that were annotated as terpenoid backbone biosynthesis genes in *S. barbata*, 32 of which had full length cDNAs (Table S5).

**Figure 3:**
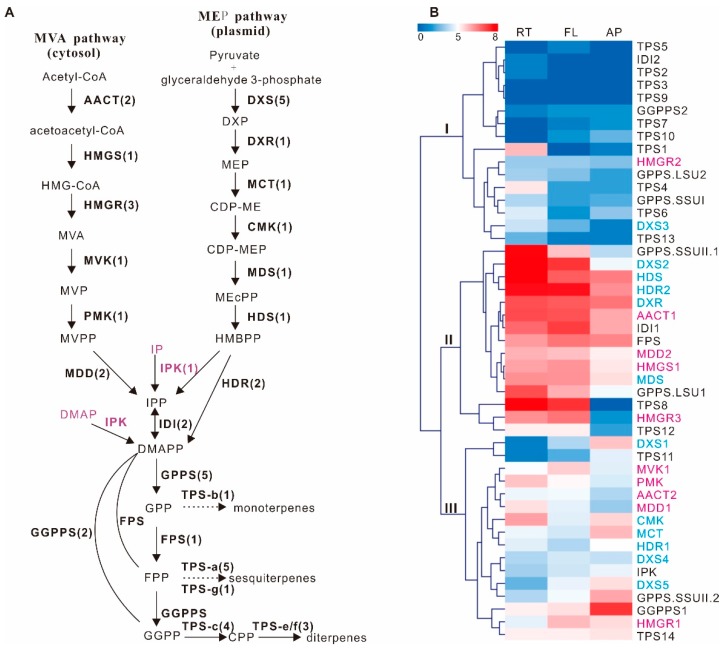
Schematics of the putative terpenoid biosynthetic pathway and the expression level of identified genes from *S. barbata*. (**A**) the putative terpenoid biosynthetic pathway in *S. barbata.* Numbers in the brackets indicate the gene family numbers of each enzyme. (**B**) a clustered heat map for the 33 terpenoid backbone genes and 14 TPS genes with log-2 transformed FPKM value in root (RT), flower (FL), and aerial parts (AP). Purple marked enzymes are involved in the MVA pathway, and blue marked enzymes are involved in the MEP pathway. The newly identified enzyme IPK in alternative MVA pathway is highlighted in purple. Abbreviations: AACT, aceto-acetyl-CoA thiolase; CMK, 4-(cytidine 5′-diphospho)-2-*C*-methyl-d-erythritol kinase; DXS, 1-deoxy-d-xylulose 5-phosphate synthase; DXR, 1-deoxy-d-xylulose-5-phosphate reductoisomerase; FPS, Farnesyl pyrophosphate synthase; HDR, (E)-4-hydroxy-3-methylbut-2-enyl diphosphate reductase; HDS, (E)-4-hydroxy-3-methylbut-2-enyl diphosphate synthase; HMGS, 3-hydroxy-3-methylglutaryl-CoA synthase; HMGR, 3-hydroxy-3-methylglutaryl-CoA reductase; GGPPS, Geranylgeranyl pyrophosphate synthase; MCT, 2-*C*-methyl-d-erythritol 4-phosphate cytidylyltransferase; IDI, Isopentenyl diphosphate isomerase; IPK, Isopentenyl phosphate kinase; MDS,2-*C*-methyl-d-erythritol 2,4-cyclodiphosphate synthase; MDD, Mevalonate diphosphate decarboxylase; MDS, 2-*C*-methyl-d-erythritol 2,4-cyclodiphosphate synthase; MVK, mevalonate kinase; Phos, phosphatase(s); PMK, phosphomevalonate kinase; TPS, terpene synthases (including monoterpene synthases and sesquiterpene synthases); CPS, copalyl diphosphate synthase; KSL, Kaurene synthase. (**A**) shows (**B**) shows.

We obtained nearly all known terpenoid backbone genes compared to *Sa. miltiorrhiza* (Figure 3A; Table S5). *Sa. mitiorrhiza* is a Lamiaceae species with available genome sequences [35], thus giving us an opportunity to investigate the species-specific evolution of these genes. Some gene expansions were observed in these two species and they have the same gene families except for MDD and HMGR. In *S. barbata*, MDD form a small gene family that contains two members compared with the single gene in *Sa. miltiorrhiza,* while HMGR was encoded by a smaller gene family containing only three genes in *S. barbata* compared with the four genes in *Sa. miltiorrhiza*. Genes in *S. barbata* have similarities with genes in *Sa. miltiorrhiza* from 79.9% to 97%.

In order to uncover possible biological functions and the relationship between these genes, hierarchical clustering based on the log-2 transformed FPKM value in root, flower, and aerial parts was performed. The genes were divided into three distinct groups (Figure 3B). Six genes, HMGR2 in the MVA pathway, DXS3 in the DXP pathway, and IDI2, GGPPS2, GPPS.LSU2, and GPPS.SSUI were included in group I. They had similar low expression levels in the three organs. Thirteen genes were placed in group II. Among them, five genes, DXS2, DXR2, MDS1, HDS, and HDR2 were in the DXP pathway. Four genes, AACT1, HMGS2, HMGR3, and MDD2 belonged to the MVA pathway, together with IDI1, and three prenyltransferases, FPS, GPPS.SSUII.1, and GPPS.LSU1. These genes had the highest expression level in root and flower. Fourteen genes were part of group III. Six genes, DXS1, DXS4, DXS5, MCT, CMK, and HDR1 were in the DXP pathway, while five genes, AACT2, HMGR1, MVK1, PMK, and MDD1 were in the MVA pathway. IPK and two other prenyltransferases, GPPS.SSUII.2 and GGPPS1, were also in this group. They had moderate expression levels in all three organs, and most of them had higher expression levels in aerial parts than in the root and flower.

### 2.3. Identification of 14 Terpene Synthase Genes

**Figure 4:**
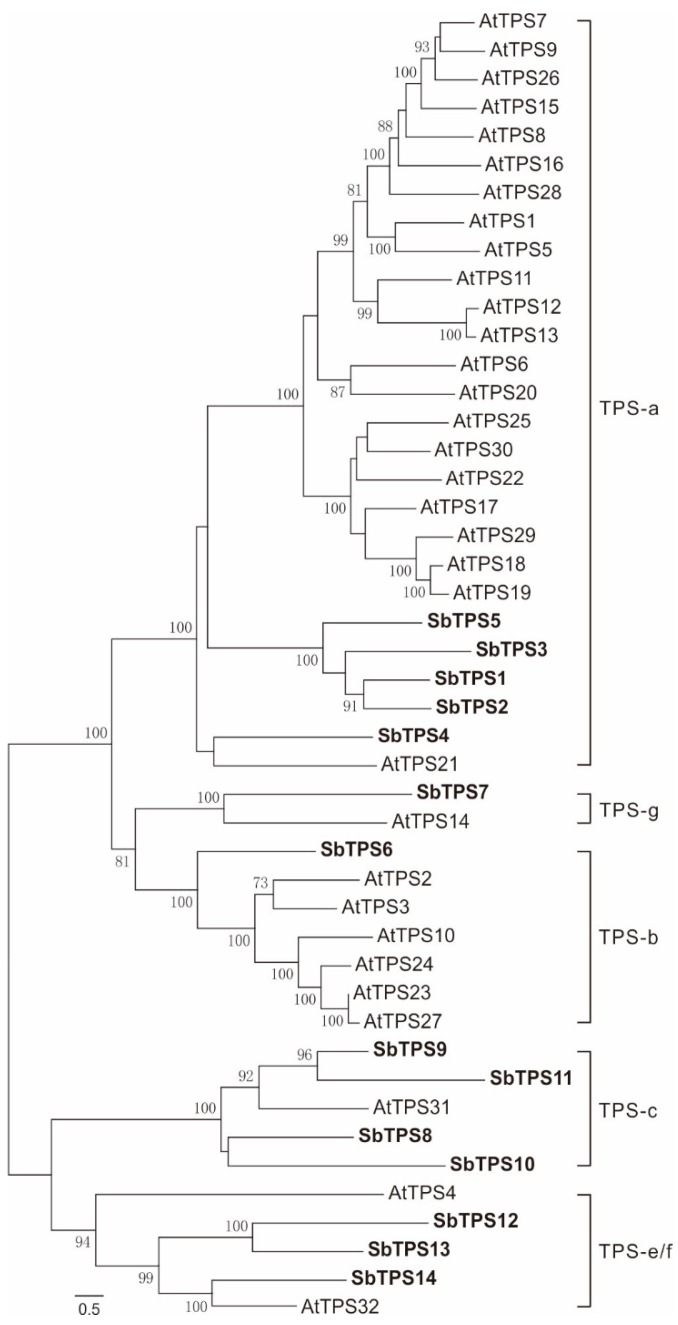
Phylogenetic tree of terpene synthase genes in *S. barbata* with those identified from *Arabidopsis thaliana*. Bold-marked enzymes show diTPS from *S. barbata* in this study.

Terpene synthases form a mid-size family that is highly diversified throughout the plant kingdom [36]. We identified 14 terpene synthases, which form a small TPS gene family (Figure 3A, Table S6). This family is even smaller than the family in *Arabidopsis thaliana*, which contains 32 TPS genes [36]. Phylogenetic analysis of the translated coding sequences placed *Sb*TPS1 to *Sb*TPS5 in the TPS-a clade (Figure 4), members of which have high similarity with gamma-cadinene synthase and germacrene D synthase (Table S6), showing that they may be responsible for sesquiterpene biosynthesis in *S. barbata*. Compared with the 22 genes in *A. thaliana,* S. *barbata* had an obvious reduction in the TPS-a subfamily. *Sb*TPS4 form a cluster with *At*TPS21, while the other four genes form a species-specific group, indicating they had different origins and different functions compared with the genes in *A. thaliana.*

*Sb*TPS6 was resolved in the TPS-b clade, which usually contains monoterpene synthases [36]. *Sb*TPS6 did not cluster with any of the TPS-b subfamily genes in *A. thaliana*. *Sb*TPS7 clustered with S-linalool synthase (*At*TPS14) in *Arabidopsis*, which is in the TPS-g subfamily. *Sb*TPS8 to *Sb*TPS11 clustered with *ent*-copalyl diphosphate synthase (AtTPS31) in *A. thaliana*, forming the TPS-c clade. Three TPS, *Sb*TPS12 to *Sb*TPS14, clustered with *ent*-kaurene synthase (*At*TPS32), which were part of TPSe/f clade (Figure 4; [36]). Despite the small gene families of TPS-a and b, *S. barbata* has a relatively large gene family in TPS-c and the TPS-e/f subfamily, which will generate structural backbones of the abundant diterpenoids in *S. barbata*.

*Sb*TPS8 and *Sb*TPS12 had similar expression patterns and were exclusively expressed in the root and flower, which was placed in group II. *Sb*TPS11 had a different expression pattern and had the highest expression in aerial parts rather than in root and flower. *Sb*TPS14 had relatively high expression in all three organs. These two genes were clustered in group III. *Neo*-clerodane diterpenoids are always isolated from the aerial parts of *S. barbata* [9,10,37] and thus *Sb*TPS11 was predicted to be involved in *neo*-clerodane diterpenoid biosynthesis in *S. barbata*.

### 2.4. Phylogenetic Relationship of diTPS within Lamiaceae

**Figure 5:**
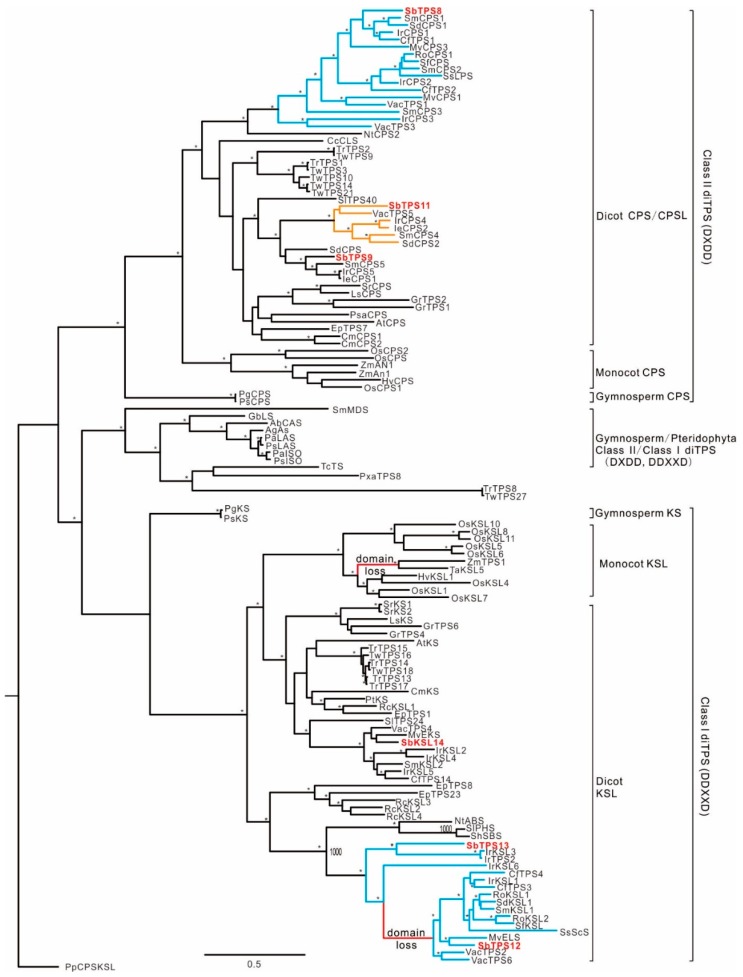
Phylogeny of *S. barbata* diterpene synthases. The maximum likelihood tree illustrates the phylogenetic relationship of *S. barbata* diterpene synthases with 127 representative characterized diTPS (Table S7). *Physcomitrella patens* CPS/kaurene (PpCPSKS) was used as an outgroup. Branches with >80% bootstrap support are indicated with an asterisk. Blue lines show diTPS genes from Lamiaceae involved in normal-CPP/LDPP-mediated diterpenoid metabolism and red lines show the loss of the N-terminal γ domain found in eudicot and monocot KSLs. Yellow lines show genes from Lamiaceae involved in the specialized *ent*-CPP/LDPP related diterpenoid metabolism. Red-marked enzymes show diTPS from *S. barbata* in this study.

Due to the abundant diterpenoids in *S. barbata*, diTPS family genes in TPS-c and TPS-e/f were studied in detail. A maximum-likelihood tree was constructed with diTPS enzymes with known functions (Table S7). *Sb*TPS8 was in the Lamiaceae-specialized CPS clade (Figure 5), together with diTPS that produce diterpenoids with “normal” stereochemical configurations [20,22,23,24,25,26,27,28,32,34] with the exception of *Sd*CPS1 from *Salvia divinorum*, which was identified to be an *ent*-CPP synthase [33]. *Sb*TPS9 and *Sb*TPS11 clustered in the *ent*-CPP synthase clade, and TPS9 was closely related to *Ie*CPS1 and *Ir*CPS1, which are involved in the gibberellin biosynthetic pathway in *Isodon eriocalyx* [21] and *I. rubescens* [32]. *Sb*TPS11 grouped into a Lamiaceae-specific clade which is responsible for specialized *ent*-kaurene, labdane, or clerodane diterpenoid formation in Lamiaceae plants [21,28,31,32,33,34]. *Sb*TPS11 was most closely related to *Vac*TPS5, sharing 64.6% sequence identity at the amino acid level. *Vac*TPS5 was identified as a kolaveny diphosphate synthase in *Vitex agnus-castus* involved in clerodane-type diterpenoid biosynthesis [34]. Thus, the relationship between *Sb*TPS11 and *Vac*TPS5 further indicated that *Sb*TPS11 is responsible for the abundant clerodane-type diterpenoid biosynthesis in *S. barbata*.

*Sb*TPS12 and *Sb*TPS13 grouped into the specialized Lamiaceae KSL clade (Figure 5). *Sb*TPS12 had the characteristic βα bidomain architecture through the loss of the γ domain at the N terminus [38]. It was most closely related to *Mv*ELS from *Marrubium vulgare* [26], sharing 72.0% sequence identity at the amino acid level. *Sb*TPS13 was most closely related to *Ir*KSL3 from *I. rubescens*, which has the common γβα tridomain structure and represents the transitional step in the evolution of Lamiaceae KSL with two domains [32]. Thus, *Sb*TPS12 and *Sb*TPS13 are predicted be involved in specialized diterpenoid biosynthesis in *S. barbata*. *Sb*TPS14 was most closely related with *Mv*EKS from *M. vulgare* [26], which was predicted to be involved in gibberellin biosynthesis.

### 2.5. Functional Characterization of Three Diterpene Synthases

**Figure 6:**
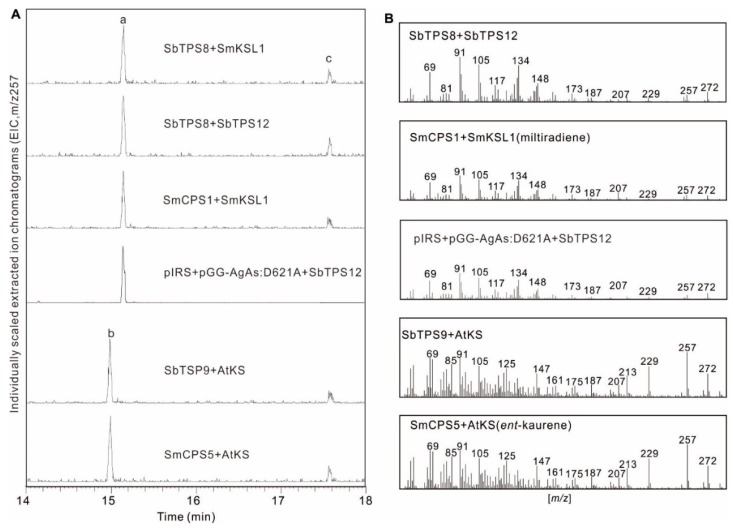
GC-MS analysis of in vitro assays with *S. barbata* diTPS. (**A**) Extracted ion chromatograms (EIC) of *m*/*z* 257 from in vitro assays with *Sb*CPS coupled with different *Sb*KSL and microbial co-expression of *Sb*TPS12. The characterized *Sm*CPS5 (*ent*-CPP synthase from *Sa. miltiorrhiza*), *Sm*CPS1 (normal-CPP synthase from *Sa. miltiorrhiza*), *Sm*KSL1 (miltiradiene synthase from *Sa. miltiorrhiza*), and *At*KS (*ent*-kaurene synthase from *A. thaliana*) were used as positive controls. a. miltiradiene; b. *ent*-kaurene; c. ent or (+)-copalol. (**B**) Corresponding mass spectra of recombinant enzyme assay products of miltiradiene and *ent*-kaurene.

Among seven identified diterpene synthase genes (four in TPS-c and three in TPS-e/f subfamily), *Sb*TPS8, *Sb*TPS9, and *Sb*TPS12 were present as full-length sequences. The open reading frames of these diTPS were directly amplified from root cDNA. The function of *Sb*TPS8, *Sb*TPS9, and *Sb*TPS12 were identified using in vitro assays with GGPP as a substrate. The product was analyzed by gas chromatography-mass spectrometry (GC-MS). The known enzymes *Sm*CPS1 (normal-CPP synthase) and *Sm*KSL1 (miltiradiene synthase) from *Sa. miltiorrhiza* produced miltiradiene [20,28], and *Sm*CPS5 (*ent*-CPP synthase) together with *At*KS (*ent*-kaurene synthase) from *A. thaliana* produced *ent*-kaurene with GGPP as substrate [32] were used as positive controls (Figure 6).

The purified recombinant *Sb*TPS8 and *Sb*TPS9 (Figure S5) individually reacted with the known enzyme of *Sm*KSL1 and *At*KS. *Sb*TPS8 in combination with *Sm*KSL1 formed miltiradiene (Figure 6), which was identified by comparison of the retention time and mass spectrum to the product of combination of *Sm*CPS1 and *Sm*KSL1 from *Sa. miltiorrhiza*. No product was observed when combining *Sb*TPS8 and *At*KS (specific to *ent*-CPP), showing that *Sb*TPS8 was a normal-copalyl diphosphate synthase. The similar combination of *Sb*TPS9 with *At*KS and *Sm*KSL1 showed that *Sb*TPS9 only produced *ent*-kaurene when combined with *At*KS (Figure 6); thus *Sb*TPS9 was found to be an *ent*-copalyl diphosphate synthase.

Furthermore, *Sb*TPS12 was found to produce miltiradiene when combined with *Sb*TPS8 (Figure 6), thus *Sb*TPS12 was identified to be a miltiradiene synthase in *S. barbata*. However, KSL from different origins were found to have extreme promiscuity [34,39,40]. Thus, to probe the substrate promiscuity of *Sb*TPS12, a previously reported modular metabolic engineering system was utilized [40,41]. This system enables facile co-expression of CPSs and KSLs in *E. coli* with high production. Twelve different class II diterpene cyclases with varied product outcomes [40] were assembled into the metabolic engineering system for co-expression with *Sb*TPS12. As expected, *Sb*TPS12 co-expressed with copalyl diphosphate synthase produced the same product of miltiradiene with an in vitro assay. No obvious stable activity was found with the other 11 class II diTPS synthases.

## 3. Discussion

*Scutellaria* is a useful plant genus with a broad global distribution. The transcriptome data generated in this study give us an overview of terpenoid biosynthesis in *S. barbata*. Thirty-three genes encode all known enzymes involved in terpenoid backbone biosynthesis and 14 terpene synthase genes were identified. Three diterpene synthase genes were further cloned from the root and functionally identified. Consistent with their close phylogenetic relationship with known diterpene synthases from Lamiaceae, *Sb*TPS8 and *Sb*TPS12 were found to produce miltiradiene, and *Sb*TPS9 would be involved in gibberellin biosynthetic pathway in *S. barbata.* Results of this study showed that besides the well-known *neo*-clerodane diterpenoids, normal-CPP mediated abietane diterpenoid biosynthesis also exists in *S. barbata*.

The size of most gene families in *S. barbata* are similar to the other 48 Lamiaceae species [12]. Some families have a much smaller number of genes, including HMGR in the MVA pathway, DXS in the MEP pathway, and TPS genes in the TPS-a and TPS-b subfamilies that are responsible for mono- and sesquiterpene biosynthesis [36]. In Lamiaceae species, the HMGR gene family in *Hyssopus officinalis* (11), *Pogostemon cablin* (12), *Prunella vulgaris* (15), and *Thymus vulgaris* (10) are even larger than those in *Gossypium raimondii*, which was identified to have the largest HMGR gene family (nine genes) in a survey including sequences from 14 land plants [42]. The DXS gene family is also highly expanded from three to 21 in *Pogostemon cablin* [12]. The TPS gene family expansion is correlated with mono- and sesquiterpene diversity [12], thus, the contraction of TPS-a and TPS-b clade genes together with HMGR in the MVA pathway may coincide with the lower chemical diversity of those compounds in *S. barbata.* HMGR and DXS have been reported as bottleneck enzymes of the MVA and MEP pathways, respectively [43]. DXS is the first step in the MEP pathway, which catalyzes pyruvate and glyceraldehyde 3-phosphate in the formation of deoxyxylulose 5-phosphate. Multiple studies have shown that DXS plays a central role in modulating the production of MEP-derived precursors to plastidial isoprenoids such as carotenoids, chlorophylls [44,45,46], and tanshinones in *Sa. Miltiorrhiza* [47,48,49]. HMGR catalyzes the conversion of HMG-CoA to MVA, which is widely used as the metabolic engineering target for improving terpenoid production [49,50,51]. The hierarchical clustering of gene expression demonstrated that genes in the MVA and MEP pathways should have different biological roles. Most of the genes in the DXP pathway (five of the seven enzymes) and MVA pathway (four of the six enzymes) had highest expression in the root (group II, Figure 3B). This group also included the complete enzymes from IPP to the diterpene synthase *Sb*TPS8 and *S*bTPS12 (Figure 3), thus illustrating root is the most metabolically active tissue in *S. barbata* for abietane diterpenoid biosynthesis. In group III, two of the DXS genes, DXS1 and DXS 5, had higher expression level in aerial parts than in root and flower. In particular, DXS1 was closely clustered with *Sb*TPS11, while HMGR1 and GGPPS1 were closely clustered with *Sb*TPS14, thus these genes provide further direction for modulating the bioactive *neo*-clerodane or abietane diterpenoids with genetic engineering.

*Neo*-clerodane diterpenoids are signature metabolites of *Scutellaria* species. Based on the specific expression level of *Sb*TPS11, we presumed that *Sb*TPS11 will function as a kolavenyl diphosphate synthase, which is the first step toward *neo*-clerodane diterpenoids. The close relationship of *Sb*TPS11 with the identified CLPP synthase from *Salvia divinorum* [31,33] and *Vitex agnus-castus* [34] in Lamiaceae further supports this hypothesis. However, *Sb*TPS11 showed no significant phylogenetic relationship with the known CLPP synthases (*Tw*TPS10 and *Tw*TPS14) of *Tripterygium wilfordii* [39]. This result further highlights that a Lamiaceae-specific lineage is responsible for specialized *ent*-kaurene, labdane, or *neo*-clerodane diterpenoid formation in Lamiaceae plants (Figure 5; [32]). *Sb*TPS8 and *Sb*TPS12 are closely related to the Lamiaceae specialized CPS and KSL, which can act subsequently with GGPP to produce normal-CPP and miltiradiene (Figure 6). The transcription patterns of *Sb*TPS8 and *Sb*TPS12 were similar to those involved in tanshinone biosynthesis in *Sa. miltiorrhiza* in the root [28]. However, *Sb*TPS8 and *Sb*TPS12 also have a higher expression level in flowers, illustrating their different physiological functions in *S. barbata*.

## 4. Materials and Methods

### 4.1. Plant Materials

*S. barbata* plants were collected in the herbal garden at Anhui University of Chinese medicine and then maintained in the greenhouse at the National Resource Center for Chinese Materia Medica, China Academy of Chinese Medical Sciences. All four tissues—namely, flower, stem, leaf, and root—were harvested. Because the whole aerial parts are used as traditional medicine, the stem and leaf were mixed as one sample for RNA extraction.

### 4.2. RNA Isolation and cDNA Library Preparation

Frozen tissues were used for RNA extraction and cDNA library preparation. Total RNAs from root, aerial parts, flower with two biological replicates were extracted using a quick RNA isolation kit (HuaYueYang biotechology, Beijing, China) according to the manufacturer’s instructions. Highly purified and intact mRNAs were enriched from total RNAs using Dynabeads^®^ mRNA purification kit (Ambion, Thermo Fisher Scientific Co. Ltd., Waltham, MA, USA). RNAs were fragmented into ~300 nt fragments by 1 min incubation at 94 °C in fragmentation buffer (10 mM ZnCl_2_, 10 mM Tris-HCl, pH 7.0). The fragmentation reaction was stopped with 50 mM EDTA, followed by standard ethanol precipitation and collected for sequencing and expression validation.

### 4.3. Illumina Sequencing

For the strand-specific RNA-seq, fragmented RNAs were re-suspended in H_2_O and used for library generation with mRNA sequencing kit (Illumina Inc. San Diego, CA, USA). Sequencing was carried out on Illumina HiSeq 4000 sequencer (Illumina Inc. San Diego, CA, USA) according to the manufacturer’s instructions and 150 nt paired-end sequencing reads were generated. Preparation and shearing of mRNA, cDNA library preparation, and sequencing were performed at Novogene (Beijing, China). The raw sequence data reported in this paper have been deposited in the Genome Sequence Archive [52] in BIG Data Center, Beijing Institute of Genomics (BIG), Chinese Academy of Sciences, under accession numbers CRA001174, that are publicly accessible at http://bigd.big.ac.cn/gsa.

### 4.4. RNA-Seq Data Processing

The quality of sequencing reads was estimated using FastQC (v0.11.3) (http://www.bioinformatics.babraham.ac.uk/projects/fastqc/). Then adaptors and low quality bases were removed using Trimmomatic (v0.33) with parameters set as “ILLUMINACLIP:adaptor.fa:2:30:10 LEADING:5 TRAILING:5 SLIDINGWINDOW:4:5 MINLEN:25” [53]. Clean reads from all samples were pooled together and fed to de novo RNA-seq assembly software Trinity (v2.2.0) with default parameters [54]. Then assembled all transcripts were used as reference and raw sequencing reads were mapped to these references using Bowtie2 with default parameters [55]. Those transcripts containing mapped reads were kept and the longest transcripts of each ‘gene’ were extracted as Unigenes. The normalized transcript expression in FPKM (Fragments Per Kilobase of transcript per Million mapped reads) were determined by the RSEM program (v1.2.19) [56]. Two replicates of each sample were treated separately and used to estimate dispersions by edgeR when performing sample normalization and differential expression genes selection. The hot map of gene expression data was generated in the MultiExperiment Viewer (MeV) 4.9.0 software [57]. The average FPKM value of the two biological replicates were log-2 transformed, hierarchical clustered with Euclidean distance.

### 4.5. Transcriptome Annotation

The open reading frame (ORF) of assembled genes and transcripts were predicted using Transdecoder (v5.0.2) (https://sourceforge.net/projects/transdecoder/) with Pfam domains [58] used as references. Only one ORF encoding the complete protein or the longest protein sequences was kept as representing ORF when multiple ones are found for each gene. Function annotation of genes and proteins were performed using Trinotate (3.1.1) (http://trinotate.github.io) integrating similarity-based search for Swiss-Prot (Blastx+blastp), TrEMBL (blastp) and Pfam (HMM) databases.

The selected terpenoid biosynthesis related genes were further manual annotated by searching the NCBI nonredundant protein sequence database using BLASTX software with default parameters. Functions were assigned based on the annotation associated with the top hit that satisfied the following criteria: (1) ≥30% sequence identity; (2) ≥30% alignment coverage of either the query or subject sequences; and (3) with BLAST E-values < 1×10^−10^. Manual assembly and alignment were carried out with Bioedit (http://www.mbio.ncsu.edu/bioedit/bioedit.html) and DNAMAN (https://www.lynnon.com/pc/framepc.html).

Gene ontology analyses were performed using in-house scripts. The significance of enrichment of DE genes in each GO term were determined at a threshold of adjusted *p*-value ≤ 0.05 using Fisher’s exact test together with Benjamini–Hochberg procedure. Visualization of enriched GO terms was generated using R and excel.

### 4.6. Phylogenetic Analysis

Protein sequence alignments were generated using MAFFT [59]. A maximum likelihood phylogenetic tree was inferred using the PhyML server [60] with four rate substitution categories, JTT substitution model, a BIONJ starting tree, and 1000 bootstrap replicates. Abbreviations and accession numbers of included diterpene synthase sequences are listed in Table S7.

### 4.7. Cloning of the Full Length DiTPS Genes

Due to the high gene expression level of diTPS genes in root, the total RNA of it was further used to verify the full length of diTPS obtained from transcriptome assembly. One to 5 μg of total RNA was reverse transcribed into cDNA using the PrimerScript^TM^ RT reagent kit with gDNA eraser (TaKaRa Corp., Dalian, China), according to the manufacturer’s instructions. A pEASY^®^-Uni Seamless Cloning and Assembly Kit (TransGen Biotech, Beijing, China) was used for directly cloning the full length cDNA into pET32 plasmid (Merck, Kenilworth, NJ, USA) for expression in *Escherichia coli*. The gene-specific oligonucleotides are shown in Table S8. The plasmid was further verified by sequencing.

### 4.8. In Vitro Assays

The recombinant diTPS proteins were expressed in *E. coli* Trans B (DE3) Competent Cells, affinity-purified, and assayed with GGPP as the substrate. *Sm*CPS1 (Genbamk: KC814639); *Sm*CPS5 (KC814642), *Sm*KSL1 (ABV08817) from *Salvia miltiorrhiza* and *At*KS (AAC39443) from *Arabidopsis thaliana* were used in this study. The expression and purification of the recombinant proteins were performed as described by Cui et al. [28]. In short, the constructs were transformed into Trans B (DE3) competent cells. Three to five positive colonies were cultured in LB medium with 50 mg/L carbenicillin, and 0.1 to 0.4 mM isopropyl-β-D-thiogalactopyranoside was added to induce the expression of the protein. Subsequently, cell pellets were collected and resuspended in assay buffer (50 mM Phosphate buffer, pH 7.4, 10% glycerol, 2 mM DTT, and 10 mM MgCl_2_) and sonicated six times for 10 s, on ice. Lysate from the samples was centrifuged at 12,000 g for 20 min at 4 °C. The proteins were purified using nickel-nitrilotriacetic acid agarose beads following the method previously described by Cui et al. [28].

The function of *Sb*TPS 8 and 9 was identified by combination with *Sm*KSL1 and *At*KS. Fifty mM GGPP (Sigma-Aldrich) together with different class II and class I diTPS was mixed and incubated for 4 h at 30 °C. Assay mixtures were extracted three times with an equal volume of hexane. The hexane fractions were pooled, evaporated under nitrogen, resuspended in 50 μL of hexane, and then analyzed by GC-MS.

### 4.9. Microbial Co-Expression of SbTPS12

The promiscuity of SbTPS12 was investigated using a previously reported modular metabolic engineering system [40]. This experiment was performed by co-transforming *E. coli* BL21(DE3)-C41 with pET32-*Sb*TPS12 and the plasmid pIRS [61] and 12 different pGG-DTCs [40]. Cultures were grown in 50 mL TB medium (pH = 7.0) at 37 °C and recombinant strains were grown under selective conditions with antibiotics at concentrations of 25 μg/mL for carbenicillin, 20 μg/mL for chloramphenicol, and 15 μg/mL for spectinomycin. These cultures were first grown at 37 °C to mid-log phase (OD600 ~ 0.7), then the temperature was dropped to 16 °C for 0.5 h prior to induction with 1 mM isopropylthiogalactoside (IPTG) and supplementation with 40 mM pyruvate and 1 mM MgCl_2_. The induced cultures were grown for an additional 72 h before extraction with an equal volume of hexanes, the organic phase was then separated and concentrated under nitrogen and finally resuspended in 50 μL of hexane then analyzed by GC-MS.

### 4.10. Diterpene Analysis Using GC-MS

The assay was carried out using a Trace 1310 series GC with a TSQ8000 MS detector (Thermo Fisher Scientific Co. Ltd., Waltham, Massachusetts, MA, USA). A 1 μL portion of extract was injected in splitless mode onto the column. Chromatographic separation was performed on a TR-5ms capillary column (30 m × 0.25 mm ID; DF = 0.25 μm; Thermo Fisher Scientific Co. Ltd., Waltham, MA, USA). Helium was used as the carrier gas at a constant flow rate of 1 mL/min through the column. The injector temperature was set at 280 °C and the injector temperature was 280 °C. The oven program was as follows: 50 °C for 2 min, linear ramp at a rate of 20 °C·min^−1^ to 200 °C, followed with a linear ramp at a rate of 5 °C·min^−1^ to 300 °C, held at 300 °C for 10 min. The transfer line temperature was 280 °C. The data was acquired and processed using Thermo Scientific Xcalibur data handling software.

## Supplementary Materials

Figure S1: The agarose gel electrophoresis analysis of total RNA from *S. barbata*; Figure S2: Sequencing quality estimation for the six samples; Figure S3: Summary of genes annotated to Swiss-Prot, TrEMBL, and Pfam; Figure S4: Summary of Gene Ontology annotation for all UniGenes; Figure S5: The sodium dodecyl sulphate-polyacrylamide gel electrophoresis (SDS-PAGE) analysis of the recombinant of TPS8, TPS9, and TPS12; Table S1. The quality of total RNA from *S. barbata*; Table S2: Summary of sequencing reads; Table S3: Stats for all de novo assembled transcript isotig; Table S4: Annotated unigenes involved in terpenoid biosynthesis; Table S5: Genes information in diterpenoid backbone biosynthesis pathway in *S. barbata*; Table S6: Terpene synthase genes in *S. barbata*; Table S7: List of plant DiTPS involved in this paper; Table S8: Primers used in this study.
