# Supplementary material for: Transcriptomic Insight into Terpenoid Biosynthesis and Functional Characterization of Three Diterpene Synthases in Scutellaria barbata
Source: Molecules. 2018 Nov 12;23(11):2952. doi: 10.3390/molecules23112952 (PMC6278268; doi:10.3390/molecules23112952)
Supplement: Supplementary file 1 [file molecules-23-02952-s001.zip › molecules-385163-supplementary/Supplementary Material.pdf]

Supplementary

Transcriptomic Insight into Terpenoid Biosynthesis and Functional Characterization of Three Diterpene Synthases in *Scutellaria barbata*

Huabei Zhang [1,†], Baolong Jin [1,2,†], Junling Bu [1], Juan Guo [1], Tong Chen [1], Ying Ma [1], Jinfu Tang [1], Guanghong Cui [1,*] and Luqi Huang [1,2,*]

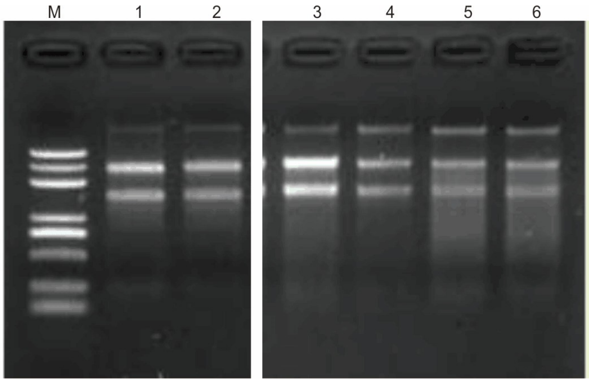

Figure S1. The agarose gel electrophoresis analysis of total RNA from *S. barbata.* M, DNA ladder; 1, aerial part 1; 2, aerial part 2; 3, root 1; 4, root 2; 5, flower 1; 6, flower 2.

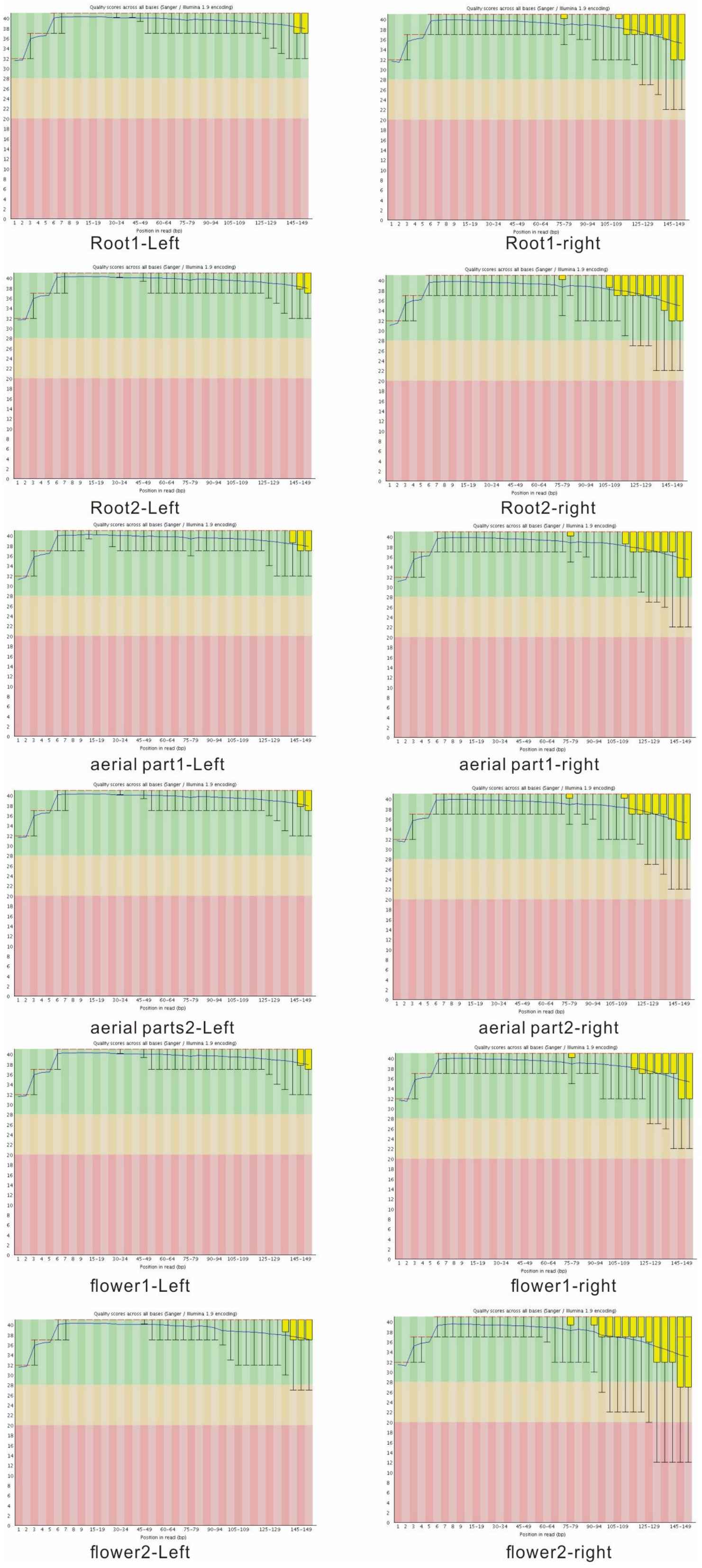

Figure S2. Sequencing quality estimation for the six samples. Each shows the Per base quality for left reads and right reads.

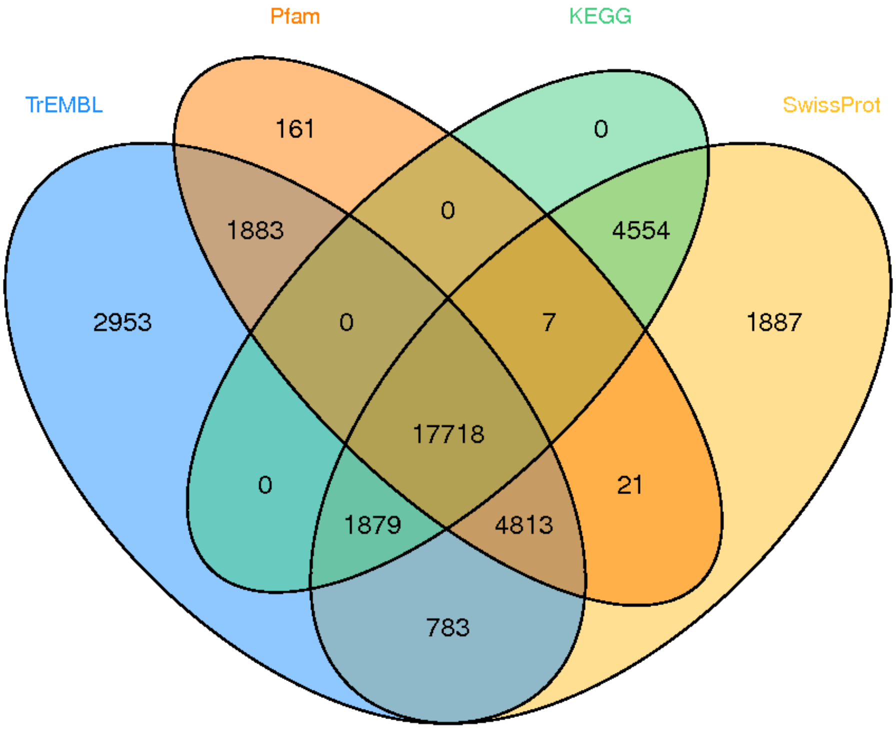

Figure S3. Summary of genes annotated to Swiss-Prot, TrEMBL and Pfam.

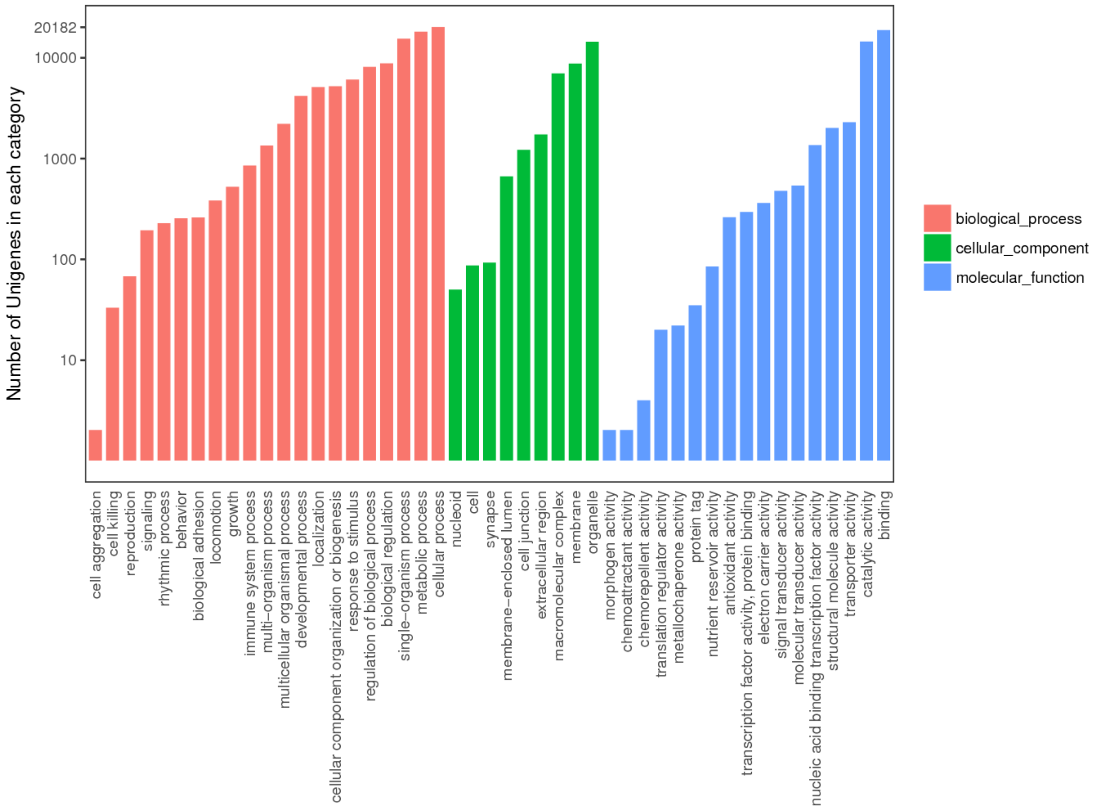

Figure S4. Summary of Gene Ontology annotation for all UniGenes

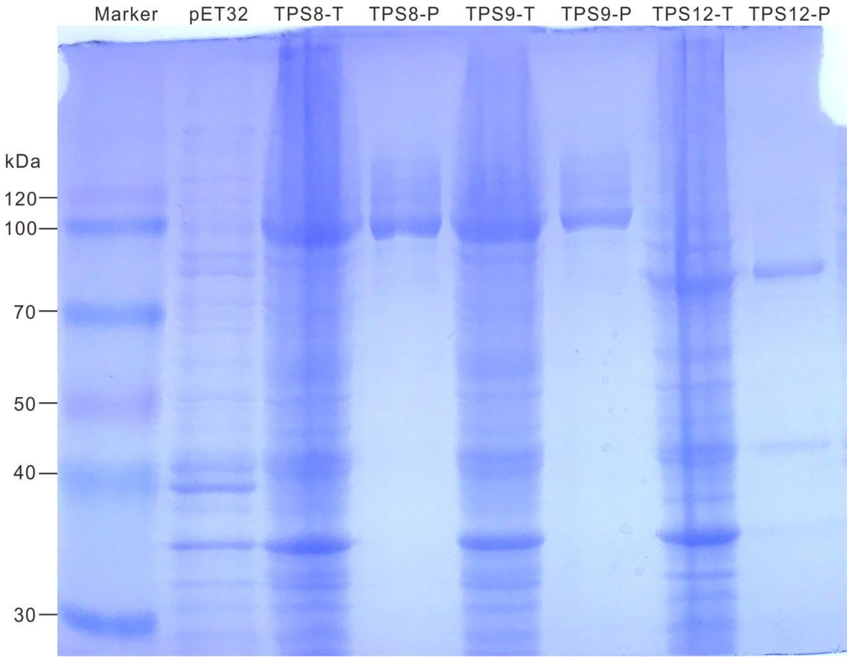

Figure S5. The sodium dodecyl sulphate-polyacrylamide gel electrophoresis (SDS-PAGE) analysis of the recombinant of TPS8, TPS9 and TPS12. T, total protein, P, purified protein using nickel-nitrilotriacetic acid agarose beads.

Table S1. The quality of total RNA from *S. barbata*

No.	Sample	conc. (ng/μl)	volume (μl)	amount(μg)	OD260/280	OD260/230	25S/18S	RIN
1	AP1	1680	18	30.24	2.09	2.393	1.5	8.9
2	AP2	1050	6	6.3	2.141	2.471	1.5	8.9
3	RT1	266	33	8.778	1.928	2.078	1.6	9.2
4	RT2	302	38	11.416	1.841	2.068	1.3	9
5	FL1	1370	21	28.77	2.213	2.445	0.6	6.5
6	FL2	1124	33	37.092	2.081	2.442	0.6	6.5

Table S2. Summary of sequencing reads (1-pair sequence left，2- pair sequence right)

Sample	Total reads	Total bases	Sequence length (nt)	GC content (%)
Root_1_1	18,171,779	2,725,766,850	150	45
Root_1_2	18,171,779	2,725,766,850	150	45
Root_2_1	20,291,672	3,043,750,800	150	45
Root_2_2	20,291,672	3,043,750,800	150	45
Aerial parts_1_1	21,428,637	3,214,295,550	150	46
aerial parts _1_2	21,428,637	3,214,295,550	150	46
aerial parts _2_1	20,110,277	3,016,541,550	150	46
aerial parts _2_2	20,110,277	3,016,541,550	150	46
Flower_1_1	18,935,106	2,840,265,900	150	45
Flower_1_2	18,935,106	2,840,265,900	150	45
Flower_2_1	19,821,205	2,973,180,750	150	45
Flower_2_2	19,821,205	2,973,180,750	150	45

Table S3. Stats for all *de novo* assembled transcript contigs. Given a set of contigs, each with its own length, the N50 length is defined as the length for which the collection of all contigs of that length or longer contains at least half of the sum of the lengths of all contigs, and for which the collection of all contigs of that length or shorter also contains at least 50% of the sum of the lengths of all contigs.

**Item**	**Value**
Total trinity 'genes'	52,211
Total trinity transcripts	88,980
GC content (%)	42.2
Stats based on ALL transcript contigs	
Contig N10 (nt)	4,534
Contig N20 (nt)	3,570
Contig N30 (nt)	2,976
Contig N40 (nt)	2,523
Contig N50 (nt)	2,144
The minimum length (nt)	201
Median contig length (nt)	1,022
Average contig length (nt)	1,370.60
Total assembled bases	121,958,564

Table S5. Genes information in diterpenoid backbone biosynthesis pathway in *S. barbata*.

Enzyme	Enzyme Name	Expression Level			Unigene (bp)	Protein (aa)	Accession No.[b]	Orthology [c]
		RT	LF	FL				
**Mevalonate Pathway (MVA Pathway)**								
AACT1	Acetyl-CoA acetyltransferase	131	63	123	2406	403	MK035027	2
AACT2		26	13	28	2223	393	MK035028	
HMGS	Hydroxymethylglutaryl-CoA synthase	66	39	74	2465	460	MK035029	1
HMGR1	Hydroxymethylglutaryl-CoA reductase	24	43	55	2353	550	MK035030	4
HMGR2		10	8	10	2058	568	MK035031	
HMGR3		76	3	96	2629	593	MK035032	
MVK	Mevalonate kinase	30	22	47	1995	389	MK035033	1
PMK	Phosphomevalonate kinase	51	20	34	2413	508	MK035034	1
MDD1	Mevalonate diphosphate decarboxylase	41	10	24	1841	421	MK035035	1
MDD2		59	37	53	2150	419	MK035036	
**Methylerythritol Pathway (MEP Pathway)**								
DXS1	1-deoxy-D-xylulose-5-phosphate synthase	2	51	13	2685	715	MK035037	5
DXS2		272	27	157	2834	726	MK035038	
DXS3		17	2	6	3744	712	MK035039	
DXS4		13	16	19	3485	684	MK035040	
DXS5		6	42	25	3121	719	MK035041	
DXR	1-deoxy-D-xylulose-5-phosphate reductoisomerase	125	95	117	2109	417	MK035042	1
MCT	2-C-methyl-D-erythritol 4-phosphate cytidylyltransferase	26	56	19	1218	307	MK035043	1
CMK	4-(cytidine-5'-diphospho)-2-C-methyl-D-erythritol kinase	67	45	22	1247	332	MK035044	1
MDS	2-C-methyl-D-erythritol 2,4-cyclodiphosphate synthase	76	42	75	1699	234	MK035045	1
HDS	(E)-4-hydroxy-3-methylbut-2-enyl diphosphate synthase	297	88	115	3271	742	MK035046	1
HDR1	(E)-4-hydroxy-3-methylbut-2-enyl diphosphate reductase	22	32	13	1896	418	MK035047	2
HDR2		197	77	193	2453	496	MK035048	
IDI1	Isopentenyl diphosphate isomerase	102	63	144	3162	293	MK035049	2
IDI2		2	0	1	1146 [a]	238	MK035050	
IPK	Isopentenyl phosphate kinase	11	25	18	2016	334	MK035051	/
**prenyltransferase**								
FPS	Farnesyl pyrophosphate synthase	70	83	96	1713	349	MK035052	1
GGPPS1	Geranylgeranyl pyrophosphate synthase	36	158	41	2231	360	MK035053	7
GGPPS2		2	3	3	1449	356	MK035054	
GPPS.SSUI		13	5	4	1331	305	MK035055	
GPPS.SSUII.1		228	14	52	2101	330	MK035056	
GPPS.SSUII.2		13	64	28	1457	363	MK035057	
GPPS.LSU1		127	28	60	1348	370	MK035058	
GPPS.LSU2		11	4	8	1556	331	MK035059	

[a] unigenes without full length cDNAs. [b] The nucleotide sequences of identified genes can be found in the GenBank/EMBL data libraries. [c] Orthology genes in *Salvia miltiorrhiza*.

Table S6. Terpene synthase genes in *S. barbata*.

Subfamily	Enzyme	Best Matches	Expression Level			Unigene (bp)	Protein (aa)	Accession No.[b]
			RT	LF	FL			
TPS-a	TPS1	gamma-cadinene synthase	55	2	1	3110	549	MK035060
	TPS2	gamma-cadinene synthase	2	0	1	1575[a]	470	MK035061
	TPS3	gamma-cadinene synthase	1	0	1	2119	599	MK035062
	TPS4	germacrene D synthase	39	4	4	2383	579	MK035063
	TPS5	germacrene D synthase	1	0	2	1845	542	MK035064
TPS-b	TPS6	(R)-limonene synthase	21	9	3	2709	590	MK035065
TPS-g	TPS7	nerolidol synthase	0	3	2	1775	469	MK035066
TPS-c	TPS8	Copalyl diphosphate synthase	214	1	160	2793	803	MK035061
	TPS9		1	1	1	2690	810	MK035061
	TP10		1	6	3	1419[a]		MK035061
	TPS11		2	23	5	2140[a]	518	MK035061
TPS-e/f	TPS12	Kaurene synthase	37	4	37	3102	594	MK035061
	TPS13		6	2	2	2749[a]	781	MK035061
	TPS14		37	40	37	4714[a]	707	MK035061

[a] unigenes without full length cDNAs. The nucleotide sequences of identified genes can be found in the GenBank/EMBL data libraries. [b] The nucleotide sequences of identified genes can be found in the GenBank/EMBL data libraries.

Table S7. List of plant DiTPS involved in this paper

Gene	Species	GenBank Accession	Funtional Annotation*	Ref.
AgAS	*Abies grandis*	AAB05407	abietadiene synthase	Vogel et al.,1996
AbCAS	*Abies balsamea*	AEL99953	cis-abienol synthase	Zerbe et al., 2012
AtCPS	*Arabidopsis thaliana*	AAA53632	*ent*-copalyldiphosphatesynthas	Sun et al., 1994
AtKSL	*A. thaliana*	AAC39443	*ent*-kaurene synthase	Yamaguchi et al., 1998
CcCLS	*Cistus creticus*	ADJ93862	copal-8-ol diphosphate synthase	Falara et al., 2010
CfTPS1	*Coleus forskohlii*	KF444506	copalyldiphosphate synthase	Pateraki et al., 2014
CfTPS14	*C. forskohlii*	AGN70881	*ent*-kaurene synthase	Zerbe et al., 2013
CfTPS2	*C. forskohlii*	KF444507	copal-8-ol diphosphate synthase	Pateraki et al., 2014
CfTPS3	*C. forskohlii*	KF444508	(13R) manoyl oxide synthase	Pateraki et al., 2014
CfTPS4	*C. forskohlii*	KF444509	miltiradiene synthase	Pateraki et al., 2014
CmCPS1	*Cucurbita maxima*	AAD04292	*ent*-copalyldiphosphate synthase	Smith et al.,1998
CmCPS2	*C. maxima*	AAD04293	*ent*-copalyldiphosphate synthase	Smith et al.,1998
CmKS	*C. maxima*	AAB39482	*ent*-kaurene synthase	Yamaguchi et al., 1996
EpTPS1	*Euphorbia peplus*	KC702395	*ent*-kaurene synthase	Zerbe et al., 2013
EpTPS23	*E. peplus*	KP889108	*Class I diTPS*	Andersen-Ranberg et al., 2016
EpTPS7	*E. peplus*	AGN70883	*ent*-copalyl diphosphate synthase	Zerbe et al., 2013
EpTPS8	*E. peplus*	KP889107	*Class I diTPS*	Andersen-Ranberg et al., 2016
GbLS	*Ginkgo biloba*	AAL09965	levopimaradiene synthase	Schepmann et al., 2001
GrTPS1	*Grindelia robusta*	AGN70886	13-labden-8,15-diol pyrophosphate synthase	Zerbe et al., 2013
GrTPS2	*G. robusta*	KR089902	labda-7,13E-dienyl diphosphate synthase	Zerbe et al., 2015
GrTPS4	*G. robusta*	KR089903	*ent*-kaurene synthase	Zerbe et al., 2015
GrTPS6	*G. robusta*	AGN70887	manoyl oxide	Zerbe et al., 2013
HvCPS	*Hordeum vulgare*	AAT49065	*ent*-copalyldiphosphate synthase	Spielmeyer et al., 2004
HvKSL1	*H. vulgare*	AAT49066	*ent*-kaurene synthase	Spielmeyer et al., 2004
IeCPS1	*Isodon eriocalyx*	AEP03177	*ent*-copalyldiphosphate synthase	Li et al., 2012
IeCPS2	*I. eriocalyx*	AEP03175	*ent*-copalyldiphosphate synthase	Li et al., 2012
IrTPS2	*I. rubescens*	KX831650	*Class I diTPS*	Pelot et al., 2017

IrCPS1	*I. rubescens*	KU180499	copalyl diphosphate synthase	Jin et al., 2017
IrCPS2	*I. rubescens*	KU180500	copalyl diphosphate synthase	Jin et al., 2017
IrCPS3	*I. rubescens*	KU180501	unknown	Jin et al., 2017
IrCPS4	*I. rubescens*	KU180502	*ent*-copalyl diphosphate synthase	Jin et al., 2017
IrCPS5	*I. rubescens*	KU180503	*ent*-copalyl diphosphate synthase	Jin et al., 2017
IrKSL1	*I. rubescens*	KU180504	miltiradiene synthase/manoyl oxide synthase	Jin et al., 2017
IrKSL2	*I. rubescens*	KU180505	*ent*-isopimaradiene synthase	Jin et al., 2017
IrKSL3	*I. rubescens*	KU180506	miltiradiene synthase/manoyl oxide synthase	Jin et al., 2017
IrKSL4	*I. rubescens*	KX580633	*ent*-atisrene synthase	Jin et al., 2017
IrKSL5	*I. rubescens*	KX580634	*ent*- kaurene synthase	Jin et al., 2017
IrKSL6	*I. rubescens*	KX580635	isopimaradiene synthase	Jin et al., 2017
LsCPS	*Lactuca sativa*	BAB12440	*ent*-kaurene synthase	Sawada et al., 2008
LsKS	*L. sativa*	BAB12441	*ent*-kaurene synthase	Sawada et al., 2008
MvCPS1	*Marrubium vulgare*	KJ584450	peregrinol diphosphate synthase	Zerbe et al., 2014
MvCPS3	*M. vulgare*	KJ584452	copalyl diphosphate synthase	Zerbe et al., 2014
MvEKS	*M. vulgare*	KJ584453	*ent*-kaurene synthase	Zerbe et al., 2014
MvELS	*M. vulgare*	KJ584454	9,13-epoxy-labd-14-en synthase	Zerbe et al., 2014
NtABS	*Nicotiana tabacum*	CD33019	cis-abienol synthase	Sallaud et al., 2012
NtCPS2	*N. tabacum*	CCD33018	copal-8-ol diphosphate synthase	Sallaud et al., 2012
OsCPS1	*Oryza sativa*	BAD42449	*ent*-copalyldiphosphate synthase	Otomo et al., 2004
OsCPS2	*O. sativa*	AAT11021	*ent*-copalyldiphosphate synthase	Prisic et al., 2004
OsCPS4	*O. sativa*	AAS98158	*syn*-copalyldiphosphate synthase	Xu et al., 2004
OsKLS8	*O. sativa*	Q6BDZ9	stemar-13-ene synthase	Nemoto et al., 2004
OsKS1	*O. sativa*	AAQ72559	*ent*-kaurene synthase	Margis-Pinheiro et al., 2005
OsKSL10	*O. sativa*	Q2QQJ5	*ent*-sandaracopimara-8(14),15-diene synthase	Peters. 2006
OsKSL11	*O. sativa*	Q1AHB2	stemod-13(17)-ene synthase	Morrone et al., 2006
OsKSL4	*O. sativa*	AAU05906	pimara-7,15-diene synthase	Wilderman et al., 2004
OsKSL5	*O. sativa*	Q6Z5J6	*ent*-pimara-8(14),15-diene synthase	Margis-Pinheiro et al., 2005
OsKSL6	*O. sativa*	A4KAG8	*ent*-isokaur-15-ene synthase	Xu et al., 2007

OsKSL7	*O. sativa*	BAC56714	*ent*-cassa-12,15-diene synthase	Cho et al., 2004
PaIso	*Picea abies*	AAS47690	isopimaradiene synthase	Martin et al., 2004
PaLAS	*P. abies*	AAS47691	levopimaradiene/abietadiene synthase	Martin et al., 2004
PgCPS	*P. glauca*	ADB55707	*ent*-copalyldiphosphate synthase	Keeling et al., 2010
PgKS	*P. glauca*	ADB55708	*ent*-kaurene synthase	Keeling et al., 2010
PpCPS/KS	*Physcomitrella patens*	BAF61135	*ent*-kaurene synthase	Hayashi et al., 2006
PsaCPS	*Pisum sativum*	AAB58822	Bifunctional *ent*-copalyldiphosphate synthase	Ait-Ali et al.,1997
PsCPS	*P. sitchensis*	ADB55709	*ent*-copalyldiphosphate synthase	Keeling et al., 2010
PsIso	*P. sitchensis*	ADZ45512	Isopimaradiene synthase	Keeling et al., 2011
PsKS	*P. sitchensis*	ADB55710	*ent*-kaurene synthase	Keeling et al., 2010
PsLAS	*P. sitchensis*	ADZ45517	Levopimaradiene/abietadiene synthase	Keeling et al., 2011
PtKS	*Populus trichocarpa*	XP_002311286	*ent*-kaurene synthase	Tuskan et al., 2006
PxaTPS8	*Pseudolarix amabilis*	KU685114	*Class I diTPS*	Mafu S et al., 2017
RcKSL1	*Ricinus communis*		*ent*-kaurene synthase	Jackson et al., 2014
RcKSL2	*R.communis*		*ent*-trachylobane synthase	Jackson et al., 2014
RcKSL3	*R.communis*		*ent*-sandaracopimaradiene synthase	Jackson et al., 2014
RcKSL4	*R.communis*		*ent*-beyerene synthase	Jackson et al., 2014
RoCPS1	*Rosmarinus officinalis*	KF805857	copalyl diphosphate synthase	Brückner et al.,2014
RoKSL1	*R. officinalis*	KF805858	miltiradiene synthase	Brückner et al.,2014
RoKSL2	*R. officinalis*	KF805859	miltiradiene synthase	Brückner et al.,2014
SdCPS	*Scoparia dulcis*	BAD91286	*ent*-copalyldiphosphate synthase	Nakagiri et al., 2005
ShSBS	*S. habrochaites*	B8XA41	santalene and bergamotene synthase	Sallaud et al., 2009
SlPHS	*Solanum lycopersicum*	ACO56896	phellandrene synthase	Schilmiller et al., 2009
SlTPS24	*S. lycopersicum*	AEP82778	*ent*-kaurene synthase	Falara et al., 2011
SlTPS40	*S. lycopersicum*	JN412074	*ent*-copalyldiphosphate synthase	Falara et al., 2011
SmCPS1	*S. miltiorrhiza*	KC814639	copalyl diphosphate synthase	Gao et al., 2009
SmCPS2	*S. miltiorrhiza*	KC814640	copalyl diphosphate synthase	Cui et al., 2015
SmCPS3	*S. miltiorrhiza*	KC814641	No function	Cui et al., 2015
SmCPS4	*S. miltiorrhiza*	KP063138	labd-13-en-8-ol diphosphate synthase	Cui et al., 2015
SmCPS5	*S. miltiorrhiza*	KC814642	*ent*-copalyldiphosphate synthase	Cui et al., 2015

SmKSL1	*S. miltiorrhiza*	ABV08817	miltiradiene synthase	Gao et al.,2009
SmKSL2	*S. miltiorrhiza*	KC814643	*ent*-kaurene synthase	Cui et al., 2015
SmMDS	*Selaginella moellendorffii*	AB668998	miltiradiene synthase	Sugai et al., 2011
SrCPS	*Stevia rebaudiana*	AAB87091	*ent*-copalyldiphosphate synthase	Richman et al., 1999
SrKS1	*S. rebaudiana*	AAD34294	*ent*-kaurene synthase	Richman et al., 1999
SrKS2	*S. rebaudiana*	AAD34295	*ent*-kaurene synthase	Richman et al., 1999
SsLPS	*Salvia sclarea*	*AET21247*	copal-8-ol diphosphate synthase	Schalk et al., 2012
SsScS	*S. sclarea*	AET21246	sclareol synthase	Schalk et al., 2012
TaKSL5	*Triticum aestivum*	BAL41692	nerolidol synthase	Hillwig et al., 2011
TcTS	*Taxus brevifolia*	AAC49310	taxadiene synthase	Wildung and Croteau.1996
TwTPS21	*T. Wilfordii*	KP889112	*Ent-copal-8-ol diphosphate synthase*	Andersen-Ranberg et al., 2016
TwTPS14	*Tripterygium wilfordii*	KP889111	*kolavenyl diphosphate synthase*	Andersen-Ranberg et al., 2016
TwTPS27	*T. Wilfordii*	KU948698	*Class I diTPS*	Hansen et al., 2017
TwTPS16	*T. Wilfordii*	KU948697	*Class I diTPS*	Hansen et al., 2017
TwTPS17	*T. Wilfordii*	KU948695	*Class I diTPS*	Hansen et al., 2017
TwTPS18	*T. Wilfordii*	KU948696	*Class I diTPS*	Hansen et al., 2017
TwTPS3	*T. Wilfordii*	KU948707	*Ent-copalyl diphosphate synthase*	Hansen et al., 2017
TwTPS9	*T. Wilfordii*	KU948709	*copalyl diphosphate synthase*	Hansen et al., 2017
TwTPS10	*T. Wilfordii*	KU948708	*kolavenyl diphosphate synthase*	Hansen et al., 2017
ZmAn1	*Zea mays*	AAA73960	*ent*-copalyldiphosphate synthase	Bensen et al., 1995
ZmAn2	*Z. mays*	AAT70083	*ent*-copalyldiphosphate synthase	Bensen et al., 1995
ZmTPS1	*Zea mays*	NP_001105097	sesquiterpene synthase	Schnee et al., 2002
SfCPS	*S. fruticosa*	KP091840	copalyl diphosphate synthase	Božić et al.,2015
SfKSL	*S. fruticosa*	KP091841	miltiradiene synthase	Božić et al., 2015
SdCPS1	*S. divinorum*	KX424876	*ent*-copalyldiphosphate synthas	Pelot et al., 2017
SdCPS2	*S. divinorum*	KX424877	clerodienyl diphosphate	Pelot et al., 2017 Chen et al., 2017
SdKSL1	*S. divinorum*	KY057342	*ent*-kaurene synthase like	Pelot et al., 2017
TrTPS1	*Tripterygium regilii*	KX533965	*Ent-CPP synthase*	Inabuy et al., 2017
TrTPS2	*Tripterygium regilii*	KX533964	*CPP synthase*	Inabuy et al., 2017

TrTPS13	*T. regilii*	KX533966	*Reacted with ent-CPP, CPP and syn-CPP*	Inabuy et al., 2017
TrTPS14	*T. regilii*	KX533967	*Ent-kaurene synthase*	Inabuy et al., 2017
TrTPS15	*T. regilii*	KX533968	ent-manool, (+)-manool, and syn-manool synthase	Inabuy et al., 2017
TrTPS8	*T. regilii*	KY856995	*Miltiradiene synthase*	Inabuy et al., 2017
VacTSP1	*Vitex agnus-castus*	MG696748	Peregrinol diphosphate synthase	Heskes et al., 2018
VacTSP2	*V. agnus-castus*	MG696749	Class I diTPS	Heskes et al., 2018
VacTSP3	*V. agnus-castus*	MG696750	Syn-copalyl diphosphate synthase	Heskes et al., 2018
VacTSP4	*V. agnus-castus*	MG696751	Kaurene synthase	Heskes et al., 2018
VacTSP5	*V. agnus-castus*	MG696752	Kolavenyl diphosphate synthase	Heskes et al., 2018
VacTSP6	*V. agnus-castus*	MG696753	Class I diTPS	Heskes et al., 2018

Functional annotation is based on the main terpenoid product(s) of recombinant enzymes expressed in *E. coli*. Many TPSs produced multiple products.

Table S8. Primers used in this study

Name	Sequence(5'to 3')
BZL-CPS1-FF	ggccatggctgatatcggaATGGGCTCTCTATCAACT
BZL-CPS1-FR	cgacggagctcgaattcgTCACATGACTGGTTCGAA
BZL-CPS2-FF	ggccatggctgatatcggaATGCCTTTCCTCCTCCCT
BZL-CPS2FR	cgacggagctcgaattcgTTATTGAACTCTTTCAAA
BZL-KSL1-FF	ggccatggctgatatcggaATGTCGGCCGGGTTAAAC
BZL-KSL1-FR	cgacggagctcgaattcgTCAGTTTTTGCTGCTAA

Supplemental References

Ait-Ali T, Swain SM, Reid JB, Sun TP, Kamiya Y. (2003). The LS locus of pea encodes the gibberellin biosynthesis enzyme *ent*-kaurene synthase A. Plant J. 11(3): 443-454.

Bensen RJ, Johal GS, Crane VC, Tossberg JT, Schnable PS, Meeley RB, Briggs SP. (1995). Cloning and characterization of the maize An1 gene. Plant Cell 7(1): 75-84.

Andersen-Ranberg J, Kongstad KT, Nielsen MT, Jensen NB, Pateraki I, Bach SS, Hamberger B, Zerbe P, Staerk D, Bohlmann J, Moler BL, and Hamberger B. (2016). Expanding the landscape of diterpene structural diversity through stereochemically controlled combinatorial biosynthesis. Angew. Chem. Int. Ed. 55:1-6.

Božić D, Papaefthimiou D, Brückner K, de Vos RC, Tsoleridis CA, Katsarou D, Papanikolaou A, Pateraki I, Chatzopoulou FM, Dimitriadou E, et al. (2015). Towards elucidating carnosic acid biosynthesis in Lamiaceae: functional characterization of the three first steps of the pathway in *Salvia fruticosa* and *Rosmarinus officinalis*. Plos One **10:** e0124106

Brückner K, Bozic D, Manzano D, Papaefthimiou D, Pateraki I, Scheler U, Ferrer A, de Vos RC, Kanellis AK, Tissier A. (2014). Characterization of two genes for the biosynthesis of abietane-type diterpenes in rosemary (*Rosmarinus officinalis*) glandular trichomes. Phytochemistry 101, 52-64.

Chen, X., Berim, A., Dayan, F. E., & Gang, D. R. (2017). A (–)-kolavenyl diphosphate synthase catalyzes the first step of salvinorin A biosynthesis in Salvia divinorum. Journal of Experimental Botany, 68(5), 1109-1122.

Cho EM, Okada A, Kenmoku H, Otomo K, Toyomasu T, Mitsuhashi W, Sassa T, Yajima A, Yabuta G, Mori K, Oikawa H, Toshima H, Shibuya N, Nojiri H, Omori T, Nishiyama M, Yamane H. (2004). Molecular cloning and characterization of a cDNA encoding *ent*-cassa-12, 15-diene synthase, a putative diterpenoidphytoalexin biosynthetic enzyme, from suspension-cultured rice cells treated with a chitin elicitor. Plant J. 37(1): 1-8.

Falara V, Pichersky E, Kanellis AK. (2010). A copal-8-ol diphosphate synthase from the angiosperm *Cistus creticus* subsp. creticus is a putative key enzyme for the formation of pharmacologically active, oxygen-containing labdane-type diterpenes. Plant physiol 154(1): 301-310.

Falara V, Akhtar TA, Nguyen TT, Spyropoulou EA, Bleeker PM, Schauvinhold I, Pichersky E. (2011). The tomato terpene synthase gene family. Plant physiol 157(2): 770-789.

Gao W, Hillwig ML, Huang L, Cui G, Wang X, Kong JQ, Yang B, Peters RJ. (2009). A functional genomics approach to tanshinone biosynthesis provides stereochemical insights. Org Lett 11(22): 5170-5173.

Hansen NL, Heskes AM, Hamberger B, Olsen CE, Hallström BM, Andersen-Ranberg J, Hamberger B (2017) The terpene synthase gene family in *Tripterygium wilfordii* harbors a labdane-type diterpene synthase among the monoterpene synthase TPS-b subfamily. Plant J 89: 429-441.

Hayashi KI, Kawaide H, Notomi M, Sakigi Y, Matsuo A, Nozaki H. (2006). Identification and functional analysis of bifunctional *ent*-kaurene synthase from the moss Physcomitrella patens. FEBS Lett 580(26): 6175-6181.

Heskes, A.M., Sundram, T.C.M., Boughton, B.A., Jensen, N.B., Hansen, N.L., Crocoll, C., Cozzi, F., Rasmussen, S., Hamberger, B., Hamberger, B., et al. (2018). Biosynthesis of bioactive diterpenoids in the medicinal plant *Vitex agnus-castus*. The Plant journal 93: 943-958.

Hillwig ML, Xu M, Toyomasu T, Tiernan MS, Wei G, Cui G, Huang LQ, Peters RJ. (2011). Domain loss has independently occurred multiple times in plant terpene synthase evolution. Plant J. 68(6): 1051-1060.

Inabuy, F., Fischedick, J. T., Lange, I., Hartmann, M., Srividya, N., & Parrish, A. N., et al. (2017). Biosynthesis of diterpenoids in tripterygium adventitious root cultures. Plant Physiology, pp.00659.2017.

Jackson AJ, Hershey DM, Chesnut T, Xu MM, Peters RJ. (2014). Biochemical characterization of the castor bean *ent*-kaurene synthase(-like) family supports quantum chemical view of diterpene cyclization. Phytochemistry 103:13-21.

Jin BL, Cui GH, Guo J, Tang JF, Duan LX, Lin HX, Shen Y, Chen T, Zhang HB, and Huang LQ. (2017). Functional diversification of kaurene synthase-like genes in *Isodon rubescens*. Plant physiol. 174: 943–955

Keeling CI, Dullat HK, Yuen M, Ralph SG, Jancsik S, Bohlmann J. (2010). Identification and functional characterization of monofunctional *ent*-copalyl diphosphate and *ent*-kaurene synthases in white spruce reveal different patterns for diterpene synthase evolution for primary and secondary metabolism in gymnosperms. Plant Physiol 152(3): 1197-1208.

Keeling CI, Weisshaar S, Ralph SG, Jancsik S, Hamberger B, Dullat HK, Bohlmann J. (2011). Transcriptome mining, functional characterization, and phylogeny of a large terpene synthase gene family in spruce (*Picea* spp.). BMC Plant Boil 11(1): 43.

Li JL, Chen QQ, Jin QP, Gao J, Zhao PJ, Lu S, Zeng Y. (2012). IeCPS2 is potentially involved in the biosynthesis of pharmacologically active Isodon diterpenoids rather than gibberellin. Phytochemistry 76: 32-39.

Mafu S, Karunanithi P S, Palazzo T A, et al. Biosynthesis of the microtubule-destabilizing diterpene pseudolaric acid B from golden larch involves an unusual diterpene synthase [J]. Proceedings of the National Academy of Sciences of the United States of America, 2017:201612901.

Margis-Pinheiro M, Zhou XR, Zhu QH, Dennis ES, Upadhyaya NM. (2005). Isolation and characterization of a Ds-tagged rice (*Oryza sativa* L.) GA-responsive dwarf mutant defective in an early step of the gibberellin biosynthesis pathway. Plant Cell Rep 23(12): 819-833.

Martin DM, Fäldt J, Bohlmann J. (2004).Functional characterization of nine Norway spruce TPS genes and evolution of gymnosperm terpene synthases of the TPS-d subfamily. Plant Physiol 135(4): 1908-1927.

Morrone D, Jin Y, Xu M, Choi SY, Coates RM, Peters RJ.(2006). An unexpected diterpene cyclase from rice: functional identification of a stemodene synthase. Arch. Biochem. Biophys 448 (1-2):133-140.

Nakagiri T, Lee JB, Hayashi T. (2005). cDNA cloning, functional expression and characterization of *ent*-copalyldiphosphate synthase from *Scoparia dulcis* L. Plant Sci 169(4): 760-767.

NemotoT, Cho EM, Okada A, Okada K, Otomo K, Kanno Y, Toyomasu T, Mitsuhashi W, Sassa T, Minami E, Shibuya N, Nishiyama M, Nojiri H, Yamane H. (2004) Stemar-13-ene synthase, a diterpene cyclase involved in the biosynthesis of the phytoalexin oryzalexin S in rice. FEBS Lett 571 (1-3): 182-186.

Otomo K, Kenmoku H, Oikawa H, König WA, Toshima H, Mitsuhashi W, Yamane H, Sassa T, Toyomasu T. (2004). Biological functions of *ent*-and *syn*-copalyldiphosphate synthases in rice: key enzymes for the branch point of gibberellin and phytoalexin biosynthesis. Plant J 39(6): 886-893.

Pateraki I, Andersen-Ranberg J, Hamberger B, Heskes AM, Martens HJ, Zerbe P, Bach SS, Møller BL, Bohlmann J, Hamberger B. (2014). Manoyl oxide (13R), the biosynthetic precursor of forskolin, is synthesized in specialized root cork cells in *Coleus forskohlii*. Plant Physiol 164: 1222-1236.

Pelot KA, Mitchell R, Kwon M, Hagelthorn DM, Wardman JF, Chiang A, Bohlmann J, Ro DK,

Zerbe P. (2017). Biosynthesis of the psychotropic plant diterpene salvinorin A: Discovery and characterization of the *Salvia divinorum* clerodienyl diphosphate synthase. Plant J **89**:885-897.

Pelot K A, Hagelthorn D M, Addison J B, et al. Biosynthesis of the oxygenated diterpene nezukol in the medicinal plantIsodon rubescensis catalyzed by a pair of diterpene synthases:[J]. Plos One, 2017, 12(4):e0176507.

Prisic S, Xu M, Wilderman PR, Peters RJ. (2004). Rice contains two disparate *ent*-copalyldiphosphate synthases with distinct metabolic functions. Plant physiol 136(4): 4228-4236.

Peters, R. J.(2006) Uncovering the complex metabolic network underlying diterpenoid phytoalexin biosynthesis in rice and other cereal crop plants. Phytochemistry 67 (21):2307-2317.

Richman AS, Gijzen M, Starratt AN, Yang Z, Brandle JE. (1999). Diterpene synthesis in Stevia rebaudiana: recruitment and up - regulation of key enzymes from the gibberellin biosynthetic pathway. Plant J 19(4): 411-421.

Sallaud C, Rontein D, Onillon S, Jabes F, Duffe P, Giacalone C, Thoraval S, Escoffier C, Herbette G, Leonhardt N, Causse M, Tissier A. (2009). A novel pathway for sesquiterpene biosynthesis from Z, Z-farnesyl pyrophosphate in the wild tomato *Solanum habrochaites*. Plant Cell 21 (1):301-317.

Sallaud C, Giacalone C, Töpfer R, Goepfert S, Bakaher N, Rösti S, Tissier A. (2012). Characterization of two genes for the biosynthesis of the labdanediterpene Z - abienol in tobacco (*Nicotiana tabacum*) glandular trichomes. Plant J 72(1): 1-17.

Sawada Y, Katsumata T, Kitamura J, Kawaide H, Nakajima M, Asami T, Nakaminami K, Kurahashi T, Mitsuhashi W, Inoue Y, Toyomasu T. (2008).Germination of photoblastic lettuce seeds is regulated via the control of endogenous physiologically active gibberellin content, rather than of gibberellin responsiveness. J Exp Bot 59 (12), 3383-3393.

Schalk M, Pastore L, Mirata MA, Khim S, Schouwey M, Deguerry F, Pineda V, Rocci L, Daviet L. (2012). Toward a biosynthetic route to sclareol and amber odorants. J Am Chem Soc 134: 18900-18903.

Schepmann HG, Pang J, Matsuda S. (2001). Cloning and Characterization of *Ginkgo biloba* Levopimaradiene Synthase, Which Catalyzes the First Committed Step in Ginkgolide Biosynthesis. Arch Biochem Biophys 392(2): 263-269.

Schilmiller AL, Schauvinhold I, Larson M, Xu R, Charbonneau AL, Schmidt A, Wilkerson C, Last RL, Pichersky E. (2009). Monoterpenes in the glandular trichomes of tomato are synthesized from a neryl diphosphate precursor rather than geranyl diphosphate. Proc Natl Acad Sci USA 106(26):10865-10870.

Schnee C, Köllner TG, Gershenzon J, Degenhardt J. (2002). The maize gene terpene synthase 1 encodes a sesquiterpene synthase catalyzing the formation of (E)-β-farnesene, (E)-nerolidol, and (E, E)-farnesol after herbivore damage. Plant physiol 130(4): 2049-2060.

Smith MW, Yamaguchi S, Ait-Ali T, Kamiya Y. (1998). The first step of gibberellin biosynthesis in pumpkin is catalyzed by at least two copalyl diphosphate synthases encoded by differentially regulated genes. Plant physiol 118(4): 1411-1419.

Spielmeyer W, Ellis M, Robertson M, Ali S, Lenton JR, Chandler PM. (2004). Isolation of gibberellin metabolic pathway genes from barley and comparative mapping in barley, wheat and rice. Theor Appl Genet 109(4): 847-855.

Sugai Y, Ueno Y, Hayashi K, Oogami S, Toyomasu T, Matsumoto S, Natsume M, Nozaki H, Kawaide H. (2011). Enzymatic ^{13}C labeling and multidimensional NMR analysis of miltiradiene synthesized by bifunctional diterpene cyclase in *Selaginella moellendorffii*. J Biol Chem 86(50): 42840-42847.

Sun TP, Kamiya Y. (1994). The Arabidopsis GA1 locus encodes the cyclaseent –kaurene synthetase A of gibberellin biosynthesis. Plant Cell 6(10): 1509-1518.

Tuskan GA, Difazio S, Jansson S, Bohlmann J, Grigoriev I, Hellsten U, Putnam N, Ralph S, Rombauts S, Salamov A, et al. (2006).The genome of black cottonwood, Populus trichocarpa (Torr. & Gray) Science 313 (5793):1596-1604.

Vogel BS, Wildung MR, Vogel G, Croteau R. (1996). Abietadiene synthase from grand fir (*Abies grandis*) cDNA isolation, characterization, and bacterial expression of a bifunctionalditerpenecyclase involved in resin acid biosynthesis. J Biol Chem 271(38): 23262-23268.

Wilderman PR, Xu M, Jin Y, Coates RM, Peters RJ. (2004). Identification of *syn*-pimara-7, 15-diene synthase reveals functional clustering of terpene synthases involved in rice phytoalexin/allelochemical biosynthesis. Plant physiol 135(4): 2098-2105.

Wildung MR, Croteau R. (1996).A cDNA clone for taxadiene synthase, the diterpenecyclase that catalyzes the committed step of taxol biosynthesis. J Biol Chem 271(16): 9201-9204.

Xu MM, Hillwig ML, Prisic S, Coates RM, Peters RJ. (2004). Functional identification of rice *syn*-copalyl diphosphate synthase and its role in initiating biosynthesis of diterpenoidphytoalexin/allelopathic natural products. Plant J 39(3): 309-318.

Xu MM, Wilderman PR, Morrone D, Xu J, Roy A, Margis-Pinheiro M, Upadhyaya NM, Coates RM， Peters RJ. (2007). Functional characterization of the rice kaurene synthase-like gene family. Phytochemistry 68 (3): 312-326.

Yamaguchi S, Saito T, Abe H, Yamane H, Murofushi N, Kamiya Y. (1996). Molecular cloning and characterization of a cDNA encoding the gibberellin biosynthetic enzyme *ent*-kaurene synthase B from pumpkin (*Cucurbita maxima* L.).Plant J 10(2): 203-213.

Yamaguchi S, Sun TP, Kawaide H, Kamiya Y. (1998). The GA2 Locus of *Arabidopsis thaliana* encodes *ent*-kaurene synthase of gibberellin biosynthesis. Plant Physiol 116(4): 1271-1278.

Zerbe P, Chiang A, Yuen M, Hamberger B, Hamberger B, Draper JA, Bohlmann J. (2012). Bifunctionalcis-abienol synthase from *Abies balsamea* discovered by transcriptome sequencing and its implications for diterpenoid fragrance production. J Biol Chem 287(15): 12121-12131.

Zerbe P, Hamberger B, Yuen M, Chiang A, Sandhu H, Madilao L, Nguyen A, Hamberger B, Bach SS, Bohlmann J. (2013). Gene discovery of modular diterpene metabolism in nonmodel systems. Plant Physiol 162:1073-1091.

Zerbe P, Chiang A, Dullat H, O'Neil-Johnson M, Starks C, Hamberger B, Bohlmann J.(2014). Diterpene synthases of the biosynthetic system of medicinally active diterpenoids in Marrubium vulgare. Plant J 79(6):914-27.

Zerbe P, Rodriguez SM, Mafu S, Chiang A, Sandhu H K., O'Neil-Johnson M, Starks CM and Bohlmann J. (2015). Exploring diterpene metabolism in non-model species: transcriptome-enabled discovery and functional characterization of labda-7,13E-dienyl diphosphate synthase from *Grindelia robusta*. The Plant J. 83: 783-793.

783-793.
